# Large Clothing Size in Children Is Associated with High Body Mass Index and Clustering of Medical Comorbidities

**DOI:** 10.1155/2013/582967

**Published:** 2013-02-11

**Authors:** Olubukola O. Nafiu, Constance Burke

**Affiliations:** Section of Pediatric Anesthesiology, Department of Anesthesiology, University of Michigan, 1500 East Medical Centre Drive, Room UH 1H247, Ann Arbor, MI 48109-0048, USA

## Abstract

*Background*. Since most people are aware of their clothing size (CS), this prospective study explored the potential utility of CS as a proxy for body size and as a predictor of incident obesity-related health conditions in children. *Methods*. This was a prospective, cross-sectional study of 725 children aged 6–18 yr. We collected clinical, anthropometric, and sartorial data on all study subjects. Parents reported their children's usual CS. This was compared with US clothing chart for children. Based on this we determined whether a child's CS was appropriate or large for age. *Results*. The prevalence of overweight/obese was 31.4%. Among the study subjects, 36% usually wore large CS. Children who wore large CS were more likely to be overweight/obese compared to those in the normal CS group (OR = 5.6; 95% CI = 4.0–8.0, *P* < 0.001). Similarly, large CS was associated with higher rates of incident asthma (*P* = 0.003), obstructive sleep apnea (*P* = 0.01), habitual snoring (*P* = 0.02), and elevated preoperative blood pressure (*P* = 0.03). *Conclusion*. CS in children is associated with higher indices of adiposity and increased rates of obesity-related comorbidities.

## 1. Introduction

Obesity has reached epidemic proportions in American adults and children and in most parts of the developed world [1, 2]. Childhood obesity has indeed become one of the foremost issues in contemporary biomedical research particularly because of its importance in predicting adult overweight and obesity as well as its association with various cardiovascular risk factors [2]. The most common descriptor of obesity (used for health promotion information and risk stratification) is the body mass index (BMI), defined as an individual's weight in kilograms divided by the square of their height in meters (BMI =  kg/m^2^) [3, 4]. It is however becoming increasingly clear from clinical and epidemiologic studies that BMI may not be an accurate proxy for obesity-associated risks [5–7]. This is because BMI does not specify fat distribution and compared to other indices of adiposity, it correlates poorly with visceral (central) obesity, which tends to be more pathogenic given its close association with cardiovascular and metabolic risks [8–10]. Furthermore, central adiposity is associated with severe obstructive sleep apnea (OSA) in adults [11] and central apnea with severe nocturnal oxygenation desaturation in children [12, 13]. Due to the many limitations of BMI as a risk stratifier, other indices of adiposity are being explored.

Waist circumference (WC) measurement is the most commonly used measure of central adiposity and has become the leading complement to BMI for adiposity-related risk stratification [14, 15]. However, measurement of WC, though simple and inexpensive, is time consuming and may sometimes be socially or culturally problematic because clothes have to be removed for its accurate measurement. Additionally, in extremely obese patients, it may be impossible or very difficult to obtain accurate data. All these make it difficult to use WC measurement for large population studies.

Consequently, some investigators have suggested using clothing size (CS) as a potential surrogate marker for total body adiposity that could be just as useful as common indexes of adiposity in predicting risk. Indeed, for a long time CS has been known to correlate well with an individual's build [16]. Previous investigations in adults have established the potential usefulness of CS as a strong surrogate for obesity and especially regional adiposity [17]. Clothing size in adults has also been used as an indicator of cardiovascular risks [18]. Comparable studies exploring the potential use of CS in children as a proxy for body size or as a predictor of disease prevalence are currently unavailable. Therefore, we designed a prospective, cross-sectional study to test the hypothesis that CSs in children and common indices of adiposity are positively associated. We further hypothesized that children who wear large CS would have higher rates of medical comorbidities than their peers who wear normal CS.

## 2. Methods

Following Institutional Review Board (IRB) approval and written parental informed consent we carried out a prospective, cross-sectional observational study of children aged 6–18 yr scheduled for elective noncardiac operations at a tertiary teaching hospital. We chose a lower cut-off age of 6 yr because from our experience, anthropometric parameters are easier to measure from this age on because children are more cooperative. Other researchers have made similar observation [19].

### 2.1. Clinical, Anthropometric Measurements, and Operational Definition of Terms

Trained research assistants (RAs) collected all clinical and anthropometric data. Clothing sizes (pant size for boys and dress size for girls) were recorded in all patients. Children's usual clothing sizes were obtained from parental report and confirmed by checking the label on the clothing the child was wearing in the preoperative area. The clothing size printed on the children's clothing label was compared with published sartorial standards for children and used to determine appropriateness of CS [20].

Age in years, sex, and ethnicity were recorded in all study subjects. Height was measured to the nearest 0.1 cm using a wall-mounted stadiometer with the patients shoeless and head held in Frankfurt horizontal plane. Body weight was measured to the nearest 0.1 kg using a calibrated electronic weighing scale with patients lightly clad in hospital gowns. Neck circumference (NC) was measured with a flexible tape, with the children in the standing position, head held erect, at the level of the thyroid cartilage. Waist circumference (WC) was measured (to the nearest 0.1 cm), with the children standing, at the end of normal expiration, using a flexible tape at a point midway between the inferior margin of the lowest rib and the iliac crest. Measurements were taken with the tape snug but not compressing the skin. We then stratified children into two groups based on the presence or otherwise of abdominal obesity. Abdominal obesity was defined as age and gender-specific WC ≥ 90th percentile [19]. Similarly, presence of neck obesity was defined as NC ≥ 90th percentile for age and sex [21]. 

In addition to the primary surgical diagnoses, the presence of secondary diagnoses like diabetes (type I or II), bronchial asthma, and obstructive sleep apnea (OSA) was noted. Furthermore, based on preoperative blood pressure (BP) data, we defined normal BP as systolic and diastolic BP below the 90th percentile. Prehypertension (PHT) was defined as systolic and/or diastolic BP 90th percentile but <95th percentile or if the BP exceeds 120/80 mmHg even if it is below the 90th percentile. Hypertension (HT) indicates systolic and/or diastolic BP 95th percentile for age, sex, and height [22]. Diagnosis of bronchial asthma was based on the use of bronchodilators or on a physician diagnosis. Clinical diagnosis of OSA was based on either history of physician diagnosed OSA or severe snoring with parental report of cessation of breathing or where available, on reports of formal sleep studies. Habitual snoring was defined as parental or caregiver report of loud snoring in the child for at least 3 or more nights per week [23].

### 2.2. Statistical Analysis

Data analyses were performed with Statistical Package for the Social Sciences (SPSS for Windows version 19.0). Means and standard deviations of demographic and anthropometric variables were compared along gender lines. Continuous variables (age, height, weight, and BMI) were examined for normal distribution with the Kolmogorov-Smirnov test. We calculated BMI as weight in kilograms divided by the square of the height in meters (BMI  =  kg/m^2^) for all patients. We then transformed BMI into a categorical variable for the grouping of children into two categories thus, normal, overweight/obese. Normal BMI indicates sex-specific BMI between the 5th and 84th percentile, while high BMI (overweight/obese) indicates sex-specific BMI ≥ 85th percentile according to reference growth charts from the National Center for Health Statistics (NCHS)/Centers for Disease Control and Prevention (CDC) [24]. 

### 2.3. Relative Clothing Size

Because children's clothing sizes are usually presented with various qualifiers (such as X, T, Husky, etc.) we transformed the CS variable into a categorical variable labeled “relative CS.” This represents how far removed from age-appropriate CS the child's usual CS is (variability in the size the child is wearing and the size they ought to be wearing). We used skirt size for girls and pant size for boys. We were thus able to stratify children into 4 groups like this (0  =  at ideal CS, +1, +2 and >+2 above ideal CS). To investigate the relationship between CS and other variables, children were stratified into normal and large clothing size groups and differences between groups were compared using Pearson Chi squared test. 

We used receiver operating characteristic (ROC) analyses to explore the ability of CS to correctly identify children with high BMI. ROC curves are characteristically plotted to demonstrate the discriminatory power of a diagnostic test over the entire range of test results. A good test will have its curve skewed to the upper left corner [25]. The area under the curve (AUC) defines the diagnostic power of a test; a perfect score will have an AUC of 1, while an AUC of 0.5 means the test performs no better than chance. 

Finally, multivariate logistic regression analysis was used to calculate the adjusted odds ratio for the occurrence of clustering of obesity-associated comorbidities based on relative CS. The dependent variable was presence of one or more obesity-associated conditions (*vide supra*). The primary predictor variable was relative CS >  2+ (yes/no). Age and gender were included in the model as covariates. Model calibration was done with the Hosmer-Lemeshow test [26]. Finally, to determine whether CS was predicting the presence of these comorbidities independent of indices of adiposity, we constructed another logistic regression model to include BMI category, abdominal and neck adiposity. Odds ratio and 95% CI were calculated as before. All reported *P* values were two-tailed and statistical significance was set at *P* < 0.05.

## 3. Results

The study cohort included 725 children (mean age, 10.8 ± 3.7 years; 52% males; 82% Caucasian) 228 (31.4%) were overweight or obese. Boys were significantly older, taller, and weighed more than girls in the study cohort (Table 1). Furthermore, the proportion of relative clothing sizes were as follows: normal size  =  44.6%; +1 size = 13.0%; +2 size = 13.0%; >  +2 size =  29.4%. Children who wore large clothing size were younger, taller, and heavier than their peers with normal CS. Furthermore, all the indices of adiposity (including, BMI, WC and NC) were significantly higher in children belonging to the large CS group (Table 1). Certain obesity-associated diagnoses were more frequent in children belonging to the large CS category. For example, habitual snoring, OSA history, and incident bronchial asthma were more frequent in those who wore large CS (Table 1). The overall prevalence of one or more obesity-associated condition was 60.7%. Children in the large CS group were more likely than those in the normal CS group to have one or more obesity-associated health condition (35.6% versus 22.7%; *P* < 0.001).

**Figure 1:**
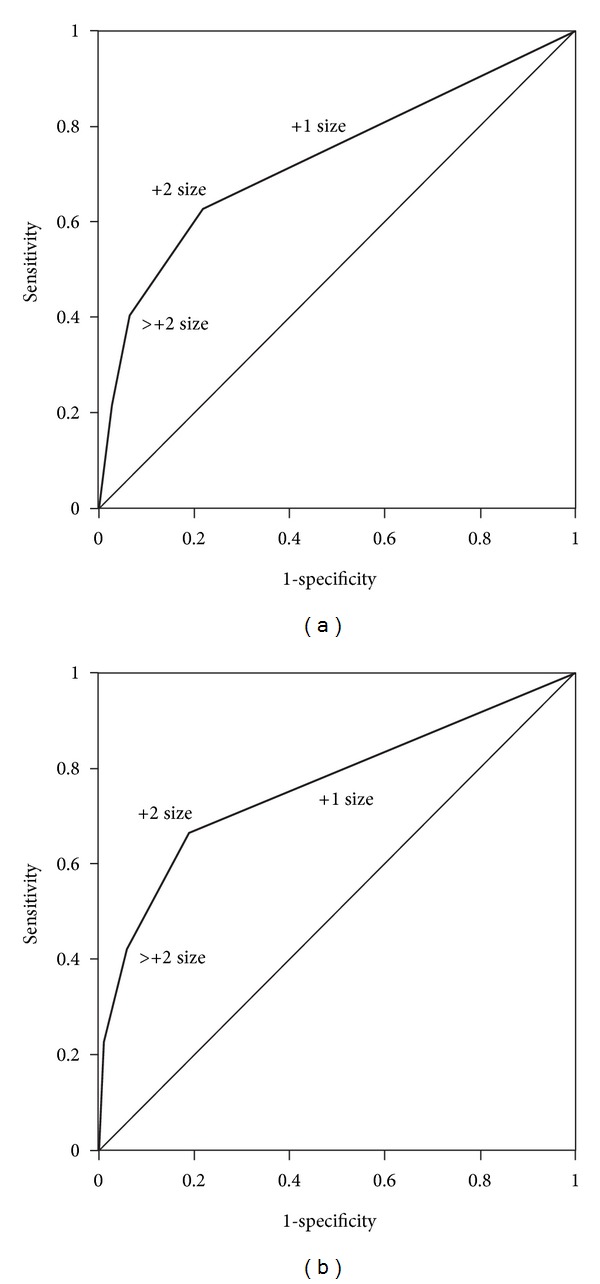
(a) ROC curve in *boys* showing the ability of relative CS to identify those with high BMI. The maximum area under the curve was at +2 size: AUC = 0.72 (95% CI = 0.69–0.77). This indicates that relative CS > +2 has good predictive ability for identifying boys with high BMI. Abbreviations: ROC: receiver operating curve; AUC = area under the curve; CS = clothing size; CI = confidence interval. (b) ROC curve in *girls* showing the ability of relative CS to identify those with high BMI. The maximum area under the curve was at +2 size: AUC = 0.76 (95% CI = 0.70–0.82). This indicates that relative CS > +2 has good predictive ability for identifying girls with high BMI. Abbreviations: ROC: receiver operating curve; AUC: area under the curve; CS: clothing size; CI: confidence interval.

In Figure 1 we present gender-specific ROC curves of the ability of relative CS to correctly identify overweight/obese children. Although the AUC was wider in females, large CS showed good discriminant ability at identifying children with high BMI in both sexes. The widest AUC for both sexes was at +2 CS, which was therefore chosen as the cut-point for categorizing the children for a logistic regression model. 

Logistic regression analyses adjusted for age and gender revealed that compared to children with relative CS less than +2, children who usually wore CS greater than +2 were 2.4 times (95% CI 1.6–3.4; *P* < 0.001) more likely to have at least one of the following obesity associated health problems: habitual snoring, OSA diagnosis, asthma diagnosis or prehypertension. Age was also a significant predictor of the occurrence of one or more of these risk factors (adjusted OR = 1.05; 95% CI = 1.01–1.13; *P* = 0.029). The Hosmer-Lemeshow goodness-of-fit test for this model was not statistically significant, indicating a good model fit (*χ* ^2^ = 5.8; df = 8; *P* = 0.665). When indices of adiposity were included in the model, the odds ratio for the association of CS with the risk factors were reduced but still remained statistically significant (Table 2). The Hosmer-Lemeshow for the expanded model showed a good model fit (*χ* ^2^= 8.81; *df* = 8; *P* = 0.358).

## 4. Discussion

In this prospective, cross-sectional study of young children and adolescents, we found that large clothing size was strongly associated with high BMI category (overweight/obesity). We also report for the first time that children who usually wore large CS for age were more likely to have some obesity-related diagnoses (including habitual snoring, OSA, bronchial asthma, and elevated blood pressure). Clothing size greater than +2 size was associated with at least two times increased odds for the presence of one or more of these obesity-related health risks. 

The notion of using clothing size to predict disease is not new. Morris et al., in 1959, observed a higher incidence of “sudden cardiac deaths” in London, England bus drivers compared to the incidence in bus conductors and those with more physically active jobs [16]. Since then, many investigators have demonstrated a close association between clothing size and cardiovascular disease [18], certain abdominopelvic malignancies [27], and diseases like hypertension, diabetes, and the metabolic syndrome [17, 27]. Our study provides the first evidence in children that it may be worthwhile to use CS as a screening tool for high BMI and clustering of obesity-related comorbidities. For example, children who usually wear clothing 2 sizes or more above what they should be wearing have a significantly increased risk of being overweight or obese. Such children also have at least a twofold increased risk of having one or more obesity-related health problems. 

As the childhood obesity epidemic continues relatively unabated, it has become increasingly clear that obesity prevention through programs that incorporate healthy diet and exercise is the best intervention for combating the epidemic. Such interventions would target children and their families to “buy into” a healthier lifestyle and maximize mutual reinforcement of healthy behaviors among family members. Many health and policy decisions concerning obesity are made using an individual's BMI. Indeed, BMI is the most widely used instrument for health promotion [10]. While many parents may not know or be able to recall their children's BMI or WC, many will be able to recall their children's clothing size (indeed all the parents/care givers in the present study were able to report their children's CS). Consequently, CS may prove to be a very useful tool for clinical screening and health promotion purposes that parents and children can easily relate to. It may also be a relatively convenient and inexpensive screening tool for large population studies in children. Future studies could explore the association of both clinical and metabolic perturbations with large clothing size in children.

## 5. Study Limitations and Strengths

This study has some limitations that should be considered in interpreting the results. It was conducted in one center, limiting the generalization of our finding to all children. However, single institution studies have the advantage of consistent, uniform; complete data collection. Additionally, due to its cross-sectional, nonrandomized, nonblinded design, the study may be subject to selection or observer bias. However, any potential bias is likely to be tempered by the large sample size and the fact that neither the parents nor the study subjects were aware of the study's aims or hypotheses.

Furthermore, although clothing sizes were obtained from parental report and confirmed by checking the label on the clothing the child was wearing, we did not systematically measure the clothes each subject was wearing. To this end, we cannot exclude differences in measurements used by different clothing designers. For example, we are aware that a size 8 garment from one designer, say, may have substantially different dimensions from another designer. However, it is unlikely that differences in clothing dimensions will affect the carefully measured anthropometric variables in the study subjects. It is also unlikely that it would have any significant effect on the clustering of obesity-related health conditions noted in this study. Finally, it is conceivable that some parents typically purchase larger clothing size for their children to “grow into.” This “reverse vanity sizing” is however more likely in younger children and it is unlikely to contribute to the higher anthropometric indices in children who usually wore large clothing sizes. 

## 6. Conclusion

Although clothing size has been used as a proxy for size and for risk stratification for chronic diseases in adults, we present the first pediatric study that confirms the potential for clothing size as size descriptor. We also present data for the first time in children, which indicates clustering of some obesity-associated health conditions in children who wear larger clothing sizes for their age and gender. These findings may have important public health utility. The use of clothing size in health promotion information in children deserves further exploration.

## Figures and Tables

**Table 1:** Baseline clinical and anthropometric characteristics of the study cohort.

Variables	All (*N* = 725)	Normal CS (*N* = 475)	Large CS (*N* = 250)	*P *
Continuous, mean (SD)				
Age (years)	10.8 ± 3.7	11.04 ± 3.8	10.3 ± 4.7	0.02
Height (cm)	145.1 ± 21.5	144.1 ± 22.5	146.9 ± 19.1	0.09
Weight (kg)	44.6 ± 21.3	41.2 ± 19.4	50.9 ± 23.1	0.001
BMI (kg/m^2^)	20.1 ± 5.8	18.7 ± 4.5	22.7 ± 7.0	0.001
WC (cm)	71.6 ± 15.8	67.8 ± 13.1	78.9 ± 17.7	0.001
NC (cm)	31.4 ± 4.7	30.8 ± 4.5	32.6 ± 4.8	0.001
SBP (mmHg)	111.7 ± 14.2	110.5 ± 13.9	113.9 ± 14.5	0.004
DBP (mmHg)	64.7 ± 9.4	64.4 ± 9.5	65.4 ± 9.1	0.192
Categorical (%)				
Overweight/obese	31.4	18.1	56.8	0.001
Abdominal obesity	23.7	16.2	38.0	0.001
Neck obesity	10.7	8.4	15.0	0.007
Prehypertension	29.7	26.7	35.2	0.018
Asthma history	20.3	17.7	25.2	0.017
Habitual snorer	31.0	27.8	37.2	0.009
OSA history	15.2	12.6	20.0	0.011

**Table 2:** Result of logistic regression to estimate the adjusted odds ratio for the association of CS with clustering of obesity-associated morbidities.

Independent predictors	OR	95% CI	*P* value
Model 1: without indices of adiposity			
CS > +2	**2.42**	**1.43**–**3.45**	**<0.001**
Age (per year)	**1.05**	**1.01**–**1.13**	**0.023**
Sex (girl versus boy)	0.79	0.59–1.06	0.471
Model 2: including indices of adiposity			
NC > 90th percentile	**2.98**	**1.44–6.14**	**0.003**
BMI > 85th percentile	**1.83**	**1.21–2.84**	**0.006**
CS > +2	**1.64**	**1.02–2.67**	**0.043**
Abdominal obesity	1.28	0.77–2.13	0.331
Gender	0.85	0.62–1.26	0.293
Age (per year)	0.84	0.62–1.15	0.183

Abbreviations: CS: clothing size; NC: neck circumference; BMI: body mass index; OR: odds ratio; CI: confidence interval.

## Abbreviations

BMI: Body mass index
CDC: Centers for disease control
NCHS: National center for health statistics
CS: Clothing size
NC: Neck circumference
WC: Waist circumference
OSA: Obstructive sleep apnea
PHT: Prehypertension
ROC: Receiver operating characteristic
AUC: Area under the curve
OR: Odds ratio.
